# Integrity at end of life in the intensive care unit: a qualitative study of nurses’ views

**DOI:** 10.1186/s13613-021-00802-y

**Published:** 2021-02-05

**Authors:** Lena Palmryd, Åsa Rejnö, Tove E. Godskesen

**Affiliations:** 1 Perioperative Medicine and Intensive Care Function, Karolinska University Hospital, 171 76 SolnaStockholm, Sweden; 2 Palliative Research Centre, Department of Health Care Sciences, Ersta Sköndal Bräcke University College, Box 11189, 100 61 Stockholm, Sweden; 3 Department of Health Sciences, University West, 461 86 Trollhättan, Sweden; 4 Department of Medicine, Skaraborg Hospital Skövde, 541 85 Skövde, Sweden; 5 Centre for Research Ethics & Bioethics, Department of Public Health and Caring Sciences, Uppsala University, BMC, Box 564, SE-751 22 Uppsala, Sweden

**Keywords:** End-of-life, Ethics, Integrity, Intensive care, Nursing care, Palliative, Privacy, Qualitative research

## Abstract

**Background:**

Integrity is a core value for delivering ethical health care. However, there is a lack of precision in defining what integrity is and how nurses understand it. In the setting of nurses caring for critically ill and dying patients in intensive care units (ICUs), integrity has not received much attention. Therefore, the aim of this study was to explore how nurses perceive and maintain the integrity of patients during end-of-life care in the ICU setting.

**Methods:**

This study had a qualitative descriptive design. Data were collected using individual semi-structured interviews with 16 intensive care nurses working at ICUs in four Swedish hospitals. The data were analysed by applying qualitative content analysis.

**Results:**

Five overall categories were explored: seeing the unique individual; sensitive to patient vulnerability; observant of patients’ physical and mental sphere; perceptive of patients’ religion and culture; and being respectful during patient encounters. Many nurses found it difficult to define integrity and to explain what respecting integrity entails in the daily care of dying patients. They often used notions associated with respect and patient-centred attitudes, such as listening and being sensitive or by trying to describe good care. Integrity was nonetheless seen as a central value for their clinical work and a precondition for ethical nursing practice. Some nurses were concerned about patient integrity, which is at risk of being “wiped out” due to the patient’s illness/injury, unfamiliarity with the ICU environment and utter dependence on others for care. Protecting patients from harm and reducing patient vulnerability were also seen as important and a way to maintain the integrity of patients.

**Conclusions:**

The study results show that even though integrity is a fundamental ethical concept and a core value in nursing, ethical codes and guidelines are not always helpful in clinical situations in the end-of-life care of ICU patients. Hence, opportunities must regularly be made available for ICU nurses to reflect on and discuss ethical issues in terms of their decision-making and behaviour.

## Background

Integrity, a fundamental ethical concept and a core value in nursing, is heavily emphasised in professional codes of ethics. The code of conduct for nurses asserts that nurses must “demonstrate professional values such as respectfulness, responsiveness, compassion, trustworthiness and integrity” [1]. The Swedish Patient Act [2] emphasises that its aim is to “strengthen and clarify the patient´s position within health care activities and to promote the integrity of patients, self-determination and participation” (Chapter 1, § 1). Hence, respect for integrity represents an essential aspect of health care and a central ethical value for nurses both in Sweden and globally.

Ethical codes and guidelines, not to mention philosophical treatises and everyday language, use the notion of integrity extensively. In legal and philosophical frameworks, integrity is closely associated with concepts such as autonomy, dignity, empowerment, self, character and social virtues [3–5]. This indicates that the notion of integrity exhibits conceptual vagueness [3, 5–7].

There are many accounts of what integrity is. Andersson, for example, argues that there are three different, partly overlapping domains of integrity: wholeness; personal sphere and character trait [3]. The first conceptual domain, *integrity as wholeness*, relates to an inner sense of being whole and is, in turn, closely related to health. To safeguard the integrity of patients, nurses must respect the uniqueness of the patient and engage in maintaining or restoring health understood as a state of wholeness (i.e. integrity), which is especially important when patients are at their most vulnerable. The conceptual domain of *integrity as a personal sphere* is associated with respecting the individual patient´s values and preferences regarding access and admittance into physical, psychological and personal spheres [c.f. 8]. In the last conceptual domain, *integrity as a character trait*, the notion primarily relates to moral integrity or virtue [3]. Possessing a good character is a valuable social trait and a moral ideal for nurses. Andersson, who focuses on respecting the patient as a person, identifies being sensitive as a character trait that is a core virtue in nursing. She stresses the importance of being sensitive to vulnerability arising from illness or dependency. In contrast to the first two domains of integrity, which describe intrinsic values pertaining to all human beings, the third domain looks at integrity as a virtue that, while often seen as intrinsic, is a trait people have more or less of, or even not at all.

Respect for integrity is especially relevant in intensive care practice since critically ill and dying patients are cared for in technological health care environments. Patients, who may be in various states of consciousness, frequently have a variety of diagnoses and their conditions are often complex and usually unstable, requiring complicated, resource-intensive treatments entailing the use of advanced technologies. Drug treatment limit patient autonomy, increasing their vulnerability. Even though medical interventions in the intensive care unit (ICU) aim to maintain vital functions and to reduce mortality and avoid morbidity, the death rate remains high [9, 10], shifting the role of ICU nurses from providing life-sustaining measures to giving end-of-life care [11].

Integrity is a core value for delivering ethical health care. However, there is a lack of precision in defining what integrity is and how nurses understand it. In the setting of nurses caring for critically ill and dying patients in ICUs, integrity has not received much attention. Research in the field describes the preservation of integrity as complex and important, while also noting that patients are deprived of their integrity, generally without further defining the concept [12–14]. Even though nurses at ICUs work closely with physicians and nurse assistants, it is nurses who are responsible in practice for carrying out the patient care; in recognising complications, administering care interventions and coordinating the work within the critical care team. As they experience the care and its impact from such a close view, interviewing them concerning their views on integrity is of particular interest. The aim of this study was to explore how nurses perceive and maintain the integrity of patients during end-of-life care in the ICU setting.

## Methods

### Design

This study used a qualitative descriptive design [15] and adapted the Standards for Reporting Qualitative Research checklist [16].

### Settings and participants

Nurses were recruited from four ICUs in Sweden: three central intensive care units (CICU) and one thorax intensive care unit (TICU) situated in four different hospitals in Sweden, whereof two were university hospitals. CICU has a broad spectrum of patients and pathology, involving acute medicine and infectious diseases, specialities of traumatology and complicated surgery and TICU hold patients with heart diseases. ICUs in Sweden employ primary nurses, which means that every patient should be assigned an individual nurse responsible for patient care in a wide sense within 24 h, ensuring that care plans are set up and updated and having dialogues with relatives. The goal is to provide highly competent nursing care at the bedside and every nurse is responsible for one or two (sometimes three) patients. Most ICUs are designed for six to ten beds, with two- or multi-bed rooms as well as single room for special occasions such as care at the end of life. Only one ICU had single rooms for all patients. All ICUs had unrestricted visiting policies which allowed relatives to visit the patient frequently.

The regime at Swedish ICUs is avoiding the use of sedatives when possible and otherwise limiting the dose and duration period to keep the patient awake but still mechanically ventilated. This means that more patients are conscious during their ICU stay and this has been proved to be important for decreased prevalence and duration of delirium, good recovery, and early mobilisation [17]. The regime also includes that every 24 h an interruption of sedatives take place where the patient can breathe spontaneously. In cases when the patient for medical reasons needs to be deeply sedated (muscle-relaxation RASS = − 4 to RASS – 5), wakeup controls are not performed.

Administrative head managers and heads at clinics at four hospitals in Sweden were contacted by e-mail with information about the study. The heads approved the study and, on behalf of the researchers, distributed a letter of invitation to nurses who met the inclusion criterion, which was having at least one year of experience as an intensive care nurse in an ICU. The nurses were asked to contact the researchers if they were interested in participating. Sixteen nurses from the four hospitals made contact and consented to participation. The sample included 16 individuals (15 women and one man) with a wide range of work experience (Table 1).

**Table 1:** Characteristics of the nurses

Nurses (*N* = 16)	
Sex	
Female	15
Male	1
Years of nursing	
Range	10–35
Mean/median	24/25
Years of intensive care nursing	
Range	1–30
Mean/median	16/20

### Data collection

Individual semi-structured interviews were used to collect data. The interview guide, which was based on the literature, expert opinion and clinical experience, comprised open-ended questions. The first author (LP) performed all of the interviews from March to June 2019. Prior to data collection, a pilot interview (included in the study) was conducted to test the semi-structured questions, leading to modifications and improvements in the interview guide [18]. The interviews took place at a dedicated room for undisturbed conversations at respective ICU and started with a general question about what integrity meant to the nurses in their daily patient care and moved on to focus on the last weeks and days before the death of a patient, for example: “Can you please tell me what you do to maintain the integrity of the person at the end of life?” Other areas of inquiry concerned their experiences with nursing activities designed to maintain integrity and situations where the integrity of the patient was threatened. The interview guide was used as a flexible tool, making it possible for participants to further elaborate on specific questions or topics and for the interviewer to pose follow-up questions, such as: “Tell me more about that.” or “Could you give me an example?” [19]. The interviews, which were conducted during working hours in offices at the ICUs, lasted an hour on average and were audio recorded before being transcribed verbatim by LP.

### Data analysis

Latent inductive content analysis was used to examine the data [20]. This is an analysis method widely used for making replicable and valid inferences from, in this case, interviews transcribed as texts, to the contexts of its use [21]. The analysis started with repeatedly listening to the interviews and reading through the transcripts several times to become familiar with the content and identify meaning units involving the research aim. Next, meaning units were marked and condensed in a shortened version with the central meaning still intact. These condensed units were then coded and linked to other codes and compared, based on differences and similarities. After that, they were grouped into preliminary categories indicating the core messages of the interviews. Data were further compared, grouped into categories and subcategories, and given descriptive labels to reflect their content. All of the authors were engaged in the data analysis and met several times to discuss the coding and the categories. The final categorisation was discussed until consensus was reached and agreed upon by all of the authors. The first author has undertaken courses in qualitative methods and has good skills in performing qualitative analysis. The second and last authors have done extensive research using qualitative methods. They also teach qualitative methodology at master level and supervise PhD students using qualitative methods. All of the authors were engaged in the data analysis and met several times to discuss the coding and the categories.

## Results

The results describe how nurses engaged with patient integrity at the end of life and how they tried their best to respect and maintain integrity in ICU care. When asked, they found it difficult to define integrity and to explicate what respecting integrity entails in the daily care of dying patients. They nonetheless repeatedly used notions associated with respect and patient-centred attitudes, such as: listening and being sensitive or they tried to describe good care.

We identified respecting patient integrity as a central theme and a fundamental precondition for the nurses to have integrity. This theme was built up by five categories: seeing the unique individual; sensitive to patient vulnerability; observant of patients’ physical and mental sphere; perceptive of patients’ religion and culture; and being respectful during patient encounters (Table 2)*.*

**Table 2:** Data categories

Categories
Seeing the unique individual
Sensitive to patient vulnerability
Observant of patients’ physical and mental sphere
Perceptive of patients’ religion and culture
Being respectful during patient encounters

### Seeing the unique individual

The nurses often equated integrity with respecting the individual and described their strong commitment to carefully seeing the patient through the entire process, from ICU admission to the process of dying. Most dying patients in the ICU are sedated and not fully oriented to time and place. In such a situation, the nurses said that it was important not to lose sight of the patient as a unique person in order to maintain the integrity of the patient:

“Respecting the person, if any preferences or information about the person have been made known via relatives or the patient, then you have to have them in mind when treating the patient (Nurse 13).”

Some nurses mentioned that it was important to see the person as unique, also at the end of life in the ICU, because patients without a voice or the opportunity to communicate risk being left out, looked at or talked to, but not communicated with. This might threaten their integrity.

One way to show respect and to cater to the integrity of the individual patient is by focusing on good, safe care. Nurses often stressed that they must be responsive to the needs of the patients to maintain good, safe care at the end of life. Many nurses also stressed that they wanted patients to be awake and involved because the patients knew themselves best.

“You tried everything, you struggled with everything, but it wasn’t possible, and the treatment was discontinued. He was sedated, but our doctor turned off his sleep medicine in consultation with the relatives and told the patient that we had tried everything possible. He understood that these were his last moments. He was allowed to hug his family. Everyone came to him, but then he decided to be sedated again. (Nurse 12).”

The close nurse–patient relationship was often based on the nurse's willingness to get to know the patient. During conversations (which often took place on night shifts), nurses gathered information about patients’ values and beliefs on dying and death. These conversations helped the nurses to perceive the patient as a person and to preserve the integrity of the patient.

Furthermore, to maintain the integrity of the patient, nurses often tried to interpret how the patient experienced the relatives who visited and, if necessary, used medical reasons to create undisturbed time for the patient. According to the nurses, good care that maintains integrity includes understanding the situation of the patient and their relationships with their dear ones. Sometimes this involved the nurses stepping in to be the patient’s voice and explain the patient’s perspective to family members:

“We’re very used to dealing with different kinds of family situations, and it’s more a rule rather than the exception that families have problems … some patients even say that they [family members] are not welcome here before they arrive … But that’s something we’re seldom aware of (Nurse 8).”

Another nurse described situations when relatives unwanted by the patient visited:

“Sometimes there is a large family that would like to visit, but I [the nurse] have no idea what their relationships are like. The patient may not be able to say: “I don’t want to deal with that aunt”, and then she is standing at their bedside, for example. This could greatly violate the integrity of the patient, or what you say (Nurse 1).”

In situations like this the nurses were powerless because they did not know what the relationship was like between the patient and the visitor and could thus not fully conclude whether integrity was at risk.

### Sensitive to patient vulnerability

Nurses saw patient vulnerability as related to the patient´s strong dependency on the staff when critically ill, their inability to meet their own basic needs and to preserve integrity. This extreme dependency was also related to being at the end of life and not being able to exercise autonomous choices directly. As a result, the nurses found that protecting patients from harm and reducing their vulnerability was important and a way to uphold integrity. Nurses mentioned both physical and emotional vulnerability and that the integrity of the patient was nearly obliterated in the ICU. One nurse said:

“I think it’s something that everyone has [integrity], more or less, but it’s something that is almost wiped out when you’re an ICU patient. They still possess their integrity of course, but the boundaries are very much … You can compare it to a yellow onion. Usually, you have several layers, and you may not want some people to come in contact with the outermost layer, while dear ones might be allowed to come closer. In the ICU as a patient you have to let go of integrity. People who don’t know you wash your genitals and you lie naked and are very exposed. Nevertheless, there’s some integrity, I think (Nurse 1).”

In some situations, nurses tried to adapt to the situation that emerged and acted as a moral agent on behalf of the patient. One nurse described a situation in which she acted resolutely to uphold integrity once she became aware of the patient’s powerlessness and dependency. In the situation she expressed a desire to protect the integrity of the patient during a clinical round since she sensed that the patient was exposed and thus acted on behalf of the patient:

“… I had a patient with a lot of hallucinations who became worried when encountering people [the patient] did not recognise. When the doctors came [to the patient’s bedside] … to give the staff a report in the evening, 15 people came in. There’s one person who reports to the 15 others [staff] who are listening. So I also drove them all out; it’s forbidden, 15 people can’t stand here, for the sake of the patient (Nurse 2).”

Nurses also described an awareness about the effects of being confined to bed, machines and tubes. Being involved in both medical and nursing care made nurses more familiar with the patient, mostly because they spend much more time with the patients than the doctors do, putting the nurses in a special situation from the perspective of integrity:

“…. I think that as an intensive care nurse I have much greater knowledge [of the patient´s needs during the ICU period] than the doctors. Mostly because I see the patient a lot more than the doctors do – I’m there all the time (Nurse 2).”

Our study indicates that the nurses spend a great deal of time protecting patients and guarding their vulnerability by creating good conditions for proper care that preserves integrity.

### Observant of patients’ physical and mental spheres

Not knowing what would happen to them, being subject to others’ care decisions and having no control over the time or place during ICU stay easily triggered feelings of anxiety and stress in the patients, both physically and mentally. Several nurses noted that the design of the ICU possibly played an important role in affecting the integrity of patients. The nurses stated that they had to remember not expose patients to fellow patients when caring for them and thus safeguarding their personal sphere.

“Yes, but integrity is all about understanding and protecting their sphere, eliminating not only physical, but also mental and emotional, exposure (Nurse 5).”

Providing good care that preserved and maintained integrity could nevertheless be difficult at the ICU due to the prominence of the technical aspects of the care and care being performed in open spaces with little privacy.

The nurses mentioned various aspects of the integrity of patients that were challenged in typical high-tech care, such as being unable to choose the care provided, or the inability to cover parts of their bodies.

“There are various types of integrity, even in the patient. So I think integrity involves not exposing people … having all these cords and stuff on them somehow infringes on their body and sphere … (Nurse 2).”

Further, nurses strived to uphold integrity by being observant of the patient´s behaviour during nursing care, a task they found challenging. This included, for example, interpreting what patients experienced as positive, e.g. when changing their position.

“So it’s a challenge to find a balance that’s right for the patient, and I think you have to try to interpret their body language. And you have to try to interpret gestures and you have to try to interpret the response you get from the patient (Nurse 6).”

Most of the nurses considered themselves aware of the patient experience in managing their personal sphere in an effort to create continued good care that maintain integrity.

### Perceptive of patients’ religion and culture

Culture and beliefs were another recurring theme that was identified in the analysis as affecting integrity. Due to migration and globalisation, the nurses increasingly encountered cultural issues involving faith and religious beliefs that they considered as central to the integrity of the dying patient and as having a significant impact on relatives. As a result, the nurses struggled to understand the patients' cultural values, preferences and beliefs. Respecting their integrity was a challenge if relatives and staff were unaware of the preferences of ICU patients at the end of life. Sometimes, taking action on the specific religious needs of patients for the sake of integrity was difficult because they were not identified until a relatively late stage in the process of dying. The nurses had consequently become more perceptive to cultural issues. For example, with the cessation of medical interventions, the nurses found that having a dialogue with relatives was essential to be sure that they had understood what was going to happen. In some cultures, ending treatment is challenging to accept:

“Well, it’s registered as life-sustaining care. The problem with this is, I believe … that we have many patients from various cultures, and in some cultures the next of kin request that the patient be given treatment, while the patient does not want [treatment]. That indeed makes the situation more complicated (Nurse 10).”

In such situations, nurses tried to show respect for the integrity of the patient by asking relatives about their culture and beliefs regarding death and dying. In some cultures, there is a strong sense of duty to look after ill family members, and the nurses were sometimes concerned about the integrity of the patient when relatives were active in their care, since the relatives and nurses have a divergent understanding of the concept of integrity. Also, there are cultural differences in terms of how many people visit the patient, which can be challenging because ICUs are not designed to receive large numbers of visitors. The following excerpt provides an example of this from a nurse who took care of a dying patient with a prominent social status in his cultural setting:

“A man from a different cultural context, a cultural clan leader of his family, suffered cardiac arrest, causing such severe injuries that he would not survive … and there were many relatives who wanted to come … the day he passed away there were so many relatives in the ICU that the police were also present … we had to close parts of the ward … neither the doctor nor I could be in the room because it was so full of people, so … we had to stand outside with the door … It was hard to protect his integrity; you just hoped he wanted to be part of what happened (Nurse 4).”

The handling of dead bodies is deeply ingrained in culture and reflects various beliefs and values. In some cultures, according to the nurses, before allowing family and friends to view the body, making the corpse more presentable is important for the patients’ integrity to be maintained. The nurses focused on being perceptive of the relatives’ wishes and values in terms of behaving professionally and were prepared to break rules to grant those wishes, for example:

“… they had one last anointing with a priest, and we broke a rule forbidding the use of lit candles in the hallways, but we allowed it because we thought it was important for the family … They went downtown and bought a suit and shoes, and they came back and dressed him. They played his favourite music. We helped and dressed him in this nice suit. So it was vastly different from what normally happens. But it felt very good afterwards. That we could help them, so the patient was not wearing a diaper under his suit. Because he had hated that (Nurse 1).”

### Being respectful during patient encounters

The nurses often mentioned that having a professional and respectful encounter with dying ICU patients and relatives was one way to maintain the integrity of patients. When a decision to switch from life-sustaining treatment to palliative care was imminent, the situation was made easier if a good relationship had been established with both the patient and the family. A good relationship between the nurse and relatives was seen as contributing to increasing the integrity of the patient, according to the nurses, because it gave relatives a greater understanding of the patient´s situation. As relatives could vary in age, ranging from young children to the elderly, a major challenge mentioned was speaking with the patient’s dear ones and helping them understand that their loved one was near death. The following excerpt is a nurse’s description of just such an encounter with a patient’s young son:

“I had a great conversation with the 10-year-old son of a terribly ill father. So, we knew it just wasn't going to happen [that he would survive] … It was probably the hardest thing I said, that "No, I don't know. We are doing everything we can, but your dad is the sickest patient in the entire hospital”. It was extremely difficult to say that (Nurse 3).”

Being engaged in the patient and the family, and at the same time maintaining a calm professional manner where integrity was safeguarded, could sometimes be demanding. Nevertheless, nurses often stressed that open and honest dialogue was important for the dying patient as it allowed the patient to participate in their care, contributing to a respectful approach to their integrity.

“… it can be good, or it can also be bad that you get so close psychologically in some way … but one aspect is that you don’t withhold from the patient what is actually happening as well (Nurse 2).”

Not all patients have close relatives, and sometimes relatives are not available in the patient’s final days. According to nurses many patients are alone, with no contact with relatives, and their loneliness often makes feelings of vulnerability even more intense. This situation could often create an environment that threatened the integrity of the patient, as one nurse explained:

“You may have relatives; you may have many relatives and not just one. If they exist, and if they want to be there (Nurse 6).”

Mostly, relatives were seen as a positive factor for protection of integrity by the nurses when caring for the dying person. The relatives were viewed as a significant resource because they are often familiar with the earlier wishes and behaviour of the patient and can share that information and interpret the patient’s behaviour for the nurses. But sometimes the relatives were seen as having a negative influence on the integrity of the patient. Some nurses were concerned that relatives did not always know what the best interests of the dying person were and sometimes acted in their own interests, thus jeopardising the integrity of the patient. In such cases, the nurses must rely on what the family tell about the patient because many patients are unable to communicate their will. Nevertheless, the nurses also used their own assessment of the patient’s needs to determine how to best maintain the integrity of patients.

“Everyone is afraid of an undignified death. But then the question is how many people have enough knowledge to be able to decide what is good and what is not good for them. It’s clear that you have to respect what people say and what people want, but at the same time we must, of course, give them correct information so that they can base their decisions on the right foundation (Nurse 16).”

When the nurses felt that the relatives did not act in the best interest of the patient, it made it harder for them to protect the integrity of the patient, contributing to an already difficult situation. However, the nurses tried to follow the family’s will to the extent possible, as what happened would form their final memories of a close relative. The purpose of the nurses was to meet the relatives where they were and, in all situations, to make an effort to contribute to providing a good memory while maintaining the integrity of the patient.

## Discussion

The aim of this study was to investigate how nurses perceive and maintain the integrity of patients during end of life in the ICU. Based on the five categories derived from the analysis, a common thread emerged, that a fundamental precondition for nurses to respect patient integrity is that the nurses must show integrity. In this way, we see a link between integrity, understood as the promotion of wholeness or respect for personal spheres, and the character trait of having integrity [3]. While many nurses had difficulties defining the meaning of integrity and explicating what respecting integrity entails in the daily care of dying patients, they often resorted to notions associated with respect and patient-centred attitudes. Their responses denoted three interpretations of integrity: wholeness, personal spheres and a character trait, that were at play. This is in accordance with previously reported findings from ICU contexts covering issues such as patient dignity [22] and therapeutic futility [12]. Both of these studies found that the integrity of nurses was vital for furthering the best interests of the patient.

In our study the nurses often mentioned principles of action, such as respecting a patient’s uniqueness and engaging in restoration of their health, often understood as a state of wholeness. As previously noted, Andersson [3] pointed out that integrity is an important character trait, a virtue, that nurses must hold as a moral ideal. Virtue ethics is often said to focus on the person, contrary to act-based theories that building on, e.g. the four moral principles of justice, autonomy, beneficence and non-maleficence, or theories based on consequentialism [23]. It is often pointed out that virtue theory involves a notion of practical wisdom, a perceptual sensitivity, which allows people to identify what the situation requires [24]. This character-based ethics implies that nurses must develop virtues and try to act as moral agents, leading them to advocate on behalf of the patient, in order to respect and uphold the integrity of the patient. Notably, nurses in this study emphasised that in many medical situations they saw themselves as advocates for the patient in maintaining the integrity of the patient.

A central and important feature for maintaining the integrity of patients was being sensitive to patients’ vulnerability and their physical and mental sphere, which is in line with findings from acute care hospital settings [25]. The nurses also strived to see the patient, as unique and whole, with a past and present, here and now, and to pay respect to their uniqueness. They were concerned when patients met a lack of respect since that could pose a threat to the integrity of the patient. This is in keeping with previous findings from both ICU and institutional care. A reduced capacity to choose, decide and take responsibility for their own lives increases the patients’ dependency on others to act on their behalf, which is also consistent with findings from other ICU settings [26]. Seeing, hearing and respecting the patient can help restore their integrity [22]. To improve the ethical care of dying patients in the ICU, nurses need to be sensitive to the vulnerable situation patients are in and to identify threats to their integrity [3]. This has also been argued in other studies showing how negative attitudes or not paying attention to patient needs threatens their integrity [22, 27]. The relationship between nurse and patient is rendered complex in the ICU, and if the staff find the relational dimension of care to be a burden, there is a risk that the dying person becomes depersonalised. The frequent use of technically advanced treatments in the ICU can also cause the nurses to distance themselves from patients, rendering them unable to maintain the integrity of the patient. This poses significant ethical challenges and adds to the difficulties identified in this study. If patients are reduced to objects of care, they can become seriously vulnerable for infringement of integrity [28]. Moen and Nåden [22] stated that a patient who experiences being treated as an object feels humiliated. The nurses tried to reach an understanding with relatives in order for them to be able to understand the situation from the patient's perspective. At other times, nurses felt that they had to protect the patient and the integrity of the patient against encroachment from relatives. This notion of nurses taking the role of patient advocate has been widely discussed. Curtin, a leading expert in nursing ethics who developed the notion of the nurse as a patient advocate [29], asserts that advocacy is usually described as defending the rights and property of others. Applied to nurse practice, Curtin found that the role of patient advocate is present “when her [the nurse’s] practice helps return a patient to independence or when her practice helps alleviate suffering or when her practice promotes respects for patients as person” [30, p.9]. In the literature, advocacy has also been defined as “being a patient representative, defending the patient’s rights and universal rights, protecting the interests of the patient, contributing to decision-making and supporting the patient’s decisions, ethical-centred skills for the ‘professional self’, and ‘being a voice for the vulnerable” [31, p.2]. These notions have the potential to help develop a more multifaceted and rich conception of what nursing practice amounts to in this context. This relates to Sorensen and Iedema [32], who mentioned that the nurse's role as patient advocate needs to be developed and strengthened to increase patient integrity. Nevertheless, patient advocacy in nursing should be addressed with some caution.^1^ If nurses strongly identify with patients suffering, this may lead to a conflict with physicians over what nurses perceive as “therapeutic obstinacy” [33]. There is also a risk that nurses speak more about their own emotions and suffering with regard to the situation, without this really corresponding to the patient's wishes. Laurent et al. [33] thus impart that it is important that these emotional attitudes be shared in the team and deliberated together.

The nurses often mentioned respect for religion and culture as a core ethical value for maintaining the integrity of patients since religious beliefs and the role they play differ widely between cultures. Nurses in this study found that this was a significant challenge at times. Nurses consider the dying patient’s particular culture or religion as important for the patient and the integrity of the patient but also for the relatives. Nurses typically found themselves in a unique situation that allowed them to integrate many aspects of patient care in terms of culturally different traditions, rituals and values related to death. They expressed feeling a sense of responsibility to be sensitive to patients’ cultural needs and beliefs in striving to protect their integrity when patients’ families step in and feel responsible for the care of their family member. Nurses in this study found that some cultures emphasise the duty to cater to sick family and friends. They found this central to the integrity of the patient and that it had a significant impact on relatives, which is why the nurses strived to understand the patients' cultural values, preferences and beliefs. Consequently, relatives were important sources of information. Many family members wanted to visit the patient and stay for hours on end. The ICUs environment is traditionally not designed to host multiple visitors simultaneously, and many visitors in the patient room can considerably disturb both the care and the integrity of the patients and of fellow patients. The nurses also emphasised that it is a challenge to respond to those relatives who wish to take care of the patient after death. A major challenge was managing the patient’s body based on the relatives’ wishes when the nurses did not know if what they were doing was in accordance with the patient´s wishes or whether they were maintaining the integrity of the patient based on the patient´s culture and wishes. Being attentive and responsive to both the integrity of patients and the needs of close relatives, even though they belong to the same culture, is a difficult task. An even greater challenges is when nurses care for patients with a cultural background different than their own at the end of life in ICU. Previous studies have also found that providing culturally competent care is a challenge [34–36].

## Strengths and limitations

Since research describing how intensive care nurses perceive and maintain the integrity of patients during end-of-life care in the ICU setting is limited, this study used a qualitative descriptive design. One strength of this study was that the interviews were held in four intensive care units, two of them university hospitals, which provided a wider, multifaceted picture. The interview data were considered to be rich but more nurses participating could have added further depth to the inquiry. Nevertheless, nurse shortage, high workloads and lack of time at ICUs affected the recruitment. The interview received data were of a good depth and gave a wide variety of descriptions of integrity. The sample was relatively homogeneous, with mostly Swedish-speaking female nurses from an urban area, which might be a limitation. The participants though covered a wide range with both short and long professional experience. Gender distribution was uneven but reflects the gender distributions among professionals in healthcare. The interviewer’s experience of working in intensive care facilitated a trustful interview situation but could also be a limitation since things could be taken for granted based on the interviewers pre-understanding of the context. The interviewer was aware of this risk and strived to be open minded in the interviews and to pose follow-up and clarifying questions to avoid jumping to conclusions. One factor contributing to trustworthiness is that all three researchers participating in the analysis are registered nurses, one in specialised intensive care and with many years of work experience, and the two others with longstanding experience from other caring contexts as well as with competence in caring ethics. Methodological trustworthiness in the study was achieved due to the commitment to the steps used in the analysis and described in the method [20, 37]. The extensive interview quotations presented allow readers to judge the accuracy of the categorisation the study applied. The results may be transferred to other ICUs and care contexts where patients with limited autonomy are cared for at end of life. Further studies, e.g. field studies, are suggested to gain a deeper understanding of how nurses perceive and maintain the integrity of patients.

### Recommendations for clinical practice

The present study has provided a more in-depth insight into how intensive care nurses perceive and maintain the integrity of patients during end-of-life care and the results have clinical implications. First, even though no recommendations for adjustments in clinical practice can be made without further research, this study has a potential to encourage nurses to take an active role in assisting patients to reorient to time and place. To communicate with sedated or nonresponsive critically ill patients in respects for the patient as a person is a way to maintaining the integrity of patients. The trends towards lighter sedation where patients are aware of the ICU environments necessitate cognitive stimulation. Therefore, when possible, nurses should strive to gather information on patients’ values and beliefs regarding death and dying, as well as their preferences and relationship with their families in an effort to uphold integrity. Second, opportunities for ethical reflection to discuss questions concerning the decision-making and behaviour of nurses, as distinct from clinical and technical questions, should be organised regularly in intensive care, especially in circumstances involving a high risk of ethical stress. Third, the findings also have implications for education and could be embedded in nursing programme curricula to help students being prepared for ethical care of dying patients.

## Conclusions

The study conclude that even though integrity is thought to be a fundamental ethical concept and core value in nursing, ethics codes and guidelines are not always helpful to nurses in clinical praxis, such as at end of life in intensive care. Our analysis showed that ICU nurses engage with the task of maintaining the integrity of patients, even though they could not provide a clear-cut definition of integrity. They found integrity to be a central value for their clinical work and maintained that integrity is a precondition for ethical nursing practice. The nurses were concerned about the integrity of patients and stated that it runs the risk of being obliterated due to the patient's illness/injury, unfamiliarity of the ICU environment and utter dependence on others for their care.

## Data Availability

The datasets used and/or analysed during the current study are available from the corresponding author on reasonable request.

## Abbreviations

ICU: Intensive care unit
RASS: Richmond Agitation Sedation Scale

## Footnotes

1 We gratefully acknowledge an anonymous reviewer for this point.
